# Supplementary material for: Intrinsic Capacity to Predict Future Adverse Health Outcomes in Older Adults: A Scoping Review
Source: Healthcare (Basel). 2023 Feb 4;11(4):450. doi: 10.3390/healthcare11040450 (PMC9957180; doi:10.3390/healthcare11040450)
Supplement: Supplementary file 1 [file healthcare-11-00450-s001.zip › healthcare-2104507-supplementary.pdf]

Table S1. The Preferred Reporting Items for Systematic reviews and Meta-Analyses extension for Scoping Reviews (PRISMA-ScR) Checklist

SECTION	ITEM	PRISMA-ScR CHECKLIST ITEM	REPORTED ON PAGE #
**TITLE**			
Title	1	Identify the report as a scoping review.	**Page 1**
**ABSTRACT**			
Structured summary	2	Provide a structured summary that includes (as applicable): background, objectives, eligibility criteria, sources of evidence, charting methods, results, and conclusions that relate to the review questions and objectives.	**Page 1**
**INTRODUCTION**			
Rationale	3	Describe the rationale for the review in the context of what is already known. Explain why the review questions/objectives lend themselves to a scoping review approach.	**Page 2-4**
Objectives	4	Provide an explicit statement of the questions and objectives being addressed with reference to their key elements (e.g., population or participants, concepts, and context) or other relevant key elements used to conceptualize the review questions and/or objectives.	**Page 4**
**METHODS**			
Protocol and registration	5	Indicate whether a review protocol exists; state if and where it can be accessed (e.g., a Web address); and if available, provide registration information, including the registration number.	**None**
Eligibility criteria	6	Specify characteristics of the sources of evidence used as eligibility criteria (e.g., years considered, language, and publication status), and provide a rationale.	**Page 5-6**
Information sources*	7	Describe all information sources in the search (e.g., databases with dates of coverage and contact with authors to identify additional sources), as well as the date the most recent search was executed.	**Page 5**
Search	8	Present the full electronic search strategy for at least 1 database, including any limits used, such that it could be repeated.	**Page 5 and Supplementary Table S2**
Selection of sources of evidence†	9	State the process for selecting sources of evidence (i.e., screening and eligibility) included in the scoping review.	**Page 6**
Data charting process‡	10	Describe the methods of charting data from the included sources of evidence (e.g., calibrated forms or forms that have been tested by the team before their use, and whether data charting was done independently or in duplicate) and any processes for obtaining and confirming data from investigators.	**Page 6-7**
Data items	11	List and define all variables for which data were sought and any assumptions and simplifications made.	**Table 1**
Critical appraisal of individual sources of evidence§	12	If done, provide a rationale for conducting a critical appraisal of included sources of evidence; describe the methods used and how this information was used in any data synthesis (if appropriate).	**None**
Synthesis of results	13	Describe the methods of handling and summarizing the data that were charted.	**Page 7**
**RESULTS**			
Selection of sources of evidence	14	Give numbers of sources of evidence screened, assessed for eligibility, and included in the review, with reasons for exclusions at each stage, ideally using a	**Page 7 and Figure 1**

SECTION	ITEM	PRISMA-ScR CHECKLIST ITEM	REPORTED ON PAGE #
		flow diagram.	
Characteristics of sources of evidence	15	For each source of evidence, present characteristics for which data were charted and provide the citations.	**Page 7-8 and Table 1**
Critical appraisal within sources of evidence	16	If done, present data on critical appraisal of included sources of evidence (see item 12).	**None**
Results of individual sources of evidence	17	For each included source of evidence, present the relevant data that were charted that relate to the review questions and objectives.	**Table 1 and Supplementary Table S3**
Synthesis of results	18	Summarize and/or present the charting results as they relate to the review questions and objectives.	**Page 9-15**
**DISCUSSION**			
Summary of evidence	19	Summarize the main results (including an overview of concepts, themes, and types of evidence available), link to the review questions and objectives, and consider the relevance to key groups.	**Page 15-19**
Limitations	20	Discuss the limitations of the scoping review process.	**Page 19-20**
Conclusions	21	Provide a general interpretation of the results with respect to the review questions and objectives, as well as potential implications and/or next steps.	**Page 20**
**FUNDING**			
Funding	22	Describe sources of funding for the included sources of evidence, as well as sources of funding for the scoping review. Describe the role of the funders of the scoping review.	**Page 20**

JBI = Joanna Briggs Institute; PRISMA-ScR = Preferred Reporting Items for Systematic reviews and Meta-Analyses extension for Scoping Reviews.

* Where *sources of evidence* (see second footnote) are compiled from, such as bibliographic databases, social media platforms, and Web sites.

† A more inclusive/heterogeneous term used to account for the different types of evidence or data sources (e.g., quantitative and/or qualitative research, expert opinion, and policy documents) that may be eligible in a scoping review as opposed to only studies. This is not to be confused with *information sources* (see first footnote).

‡ The frameworks by Arksey and O'Malley (6) and Levac and colleagues (7) and the JBI guidance (4, 5) refer to the process of data extraction in a scoping review as data charting.

§ The process of systematically examining research evidence to assess its validity, results, and relevance before using it to inform a decision. This term is used for items 12 and 19 instead of "risk of bias" (which is more applicable to systematic reviews of interventions) to include and acknowledge the various sources of evidence that may be used in a scoping review (e.g., quantitative and/or qualitative research, expert opinion, and policy document).

From: Tricco AC, Lillie E, Zarin W, O'Brien KK, Colquhoun H, Levac D, et al. PRISMA Extension for Scoping Reviews (PRISMAScR): Checklist and Explanation. Ann Intern Med. 2018;169:467–473. doi: 10.7326/M18-0850.

Table S2. Search strategies and results of nine databases

1. Pubmed (Items found: 173)	
#1	Search "Aged"[Mesh]
#2	"elderly"[Title/Abstract] OR "older"[Title/Abstract] OR "old"[Title/Abstract] OR "aged"[Title/Abstract] OR "aging"[Title/Abstract] OR "older adult*"[Title/Abstract] OR "geriatric*"[Title/Abstract] OR "senior*"[Title/Abstract] OR "middle age*"[Title/Abstract] OR "old people"[Title/Abstract]
#3	#1 OR #2
#4	intrinsic capacit*[Title/Abstract]
#5	((((Locomotion[Title/Abstract]) AND (Vitality[Title/Abstract])) AND (Cognition[Title/Abstract])) AND (Psychology[Title/Abstract])) AND (Sensory[Title/Abstract])
#6	#4 OR #5
#7	#3 AND #6
1. Chochrane Library (Items found: 130)	
#1	MeSH descriptor: [Aged] explode all trees
#2	(elderly):ti,ab,kw OR (old):ti,ab,kw OR (geriatric*):ti,ab,kw OR (aged):ti,ab,kw OR (senior*):ti,ab,kw
#3	#1 OR #2
#4	(intrinsic capacit*):ti,ab,kw
#5	(Locomotion):ti,ab,kw AND (Vitality):ti,ab,kw AND (Cognition):ti,ab,kw AND (Psychology):ti,ab,kw AND (Sensory):ti,ab,kw
#6	#4 OR #5
#7	#3 AND #6
3. Embase (Items found: 182)	
#1	'aged'/exp
#2	old:ab,ti OR older:ab,ti OR aged:ab,ti OR aging:ab,ti OR 'older adult*':ab,ti OR geriatric*:ab,ti OR senior*:ab,ti OR 'old people':ab,ti
#3	#1 OR #2
#4	'intrinsic capacit*':ab,ti
#5	locomotion:ab,ti AND vitality:ab,ti AND cognition:ab,ti AND psychology:ab,ti AND sensory:ab,ti
#6	#4 OR #5
#7	#3 AND #6
4. Web of science (Items found: 518)	
#1	TS=(ageing OR aged OR elderly OR old OR older OR older adult* OR geriatric* OR senior*)
#2	TS=(intrinsic capacit*)
#3	TS= Locomotion AND Vitality AND Cognition AND Psychology AND Sensory
#4	#2 OR #3
#5	#1 AND #4
5. CINAHL (Items found: 64)	
#1	SU" aged OR aging OR elderly OR old OR older OR older adult* OR geriatric* OR senior* OR old people "
#2	TI " aged OR aging OR elderly OR old OR older OR older adult* OR geriatric* OR senior* OR old people "
#3	AB " aged OR aging OR elderly OR old OR older OR older adult* OR geriatric* OR senior* OR old people "
#4	#1 OR #2 OR #3
#5	SU intrinsic capacit* OR AB intrinsic capacit* OR TI intrinsic capacit*
#6	TI (Locomotion AND Vitality AND Cognition AND Psychology AND Sensory) OR AB (Locomotion AND Vitality AND Cognition AND Psychology AND Sensory) OR SU (Locomotion AND Vitality AND Cognition AND Psychology AND Sensory)
#7	#5 OR #6
#8	#4 AND #7
6. CNKI (Items found:22)	
#1	SU= (老年人 OR 老人)
#2	SU=内在能力

#3	SU=活动 AND 活力 AND 心理 AND 认知 AND 感官
#4	#2 OR #3
#5	#1 AND #4
7. 万方 (Items found:287)	
#1	主题= (老年人 OR 老人)
#2	主题=内在能力
#3	主题=活动 AND 活力 AND 心理 AND 认知 AND 感官
#4	#2 OR #3
#5	#1 AND #4
8. CBM (Items found:11)	
#1	"老年人"[不加权:扩展]
#2	"老人"[常用字段:智能]
#3	#1 OR #2
#4	"内在能力"[常用字段:智能]
#5	"活动"[常用字段:智能] AND "活力"[常用字段:智能] AND "心理"[常用字段:智能] AND "认知"[常用字段:智能] AND "感官"[常用字段:智能]
#6	#3 OR #4
#5	#3 AND #6
9. VIP (Items found:9)	
#1	题名或关键词=老年人 + 老人
#2	题名或关键词=内在能力
#3	题名或关键词=活动 + 活力 + 心理+ 认知 + 感官
#4	#2 OR #3
#5	#1 AND #4

Table S3. Characteristics of the included studies

Author, year	Country/ location	Data source	Study design	Total sample	Age, mean± SD (years)	Sex, female (%)	Follow-up (years)	Setting	Outcome measures	Main results
Beard, 2019 [23]	United Kingdom	ELSA	Longitudinal study	2560	≥60	Not mentioned	1	Community	Activities of daily living (ADL); instrumental activities of daily living (IADL)	Intrinsic capacity predicted the incident loss of ADLs and IADLs.
Charles, 2020 [17]	Belgium	SENIOR	Cohort study	604	82.9±9.1	441(73.0)	3	Nursing home	Incidence of death (medical file); falls (falls and multiple falls); autonomy decline (Katz Index)	A one-unit increase in balance and nutrition decreased the probability of death by 12% (HR=0.88, 95% CI: 0.78-0.99) and 4% (HR=0.96, 95% CI: 0.93-0.99), respectively. The risk of falling decreased when there was a one-unit increase in balance performance (HR= 0.87, 95% CI: 0.79-0.96) and in the nutrition score (HR=0.96, 95% CI: 0.93-0.98). Low scores in nutrition (OR=0.86, 95% CI: 0.77-0.96) were associated with a higher probability of autonomy decline.
Chew, 2021 [24]	Asian	Not mentioned	prospective cohort study	200	67.9±7.9	137(68.5)	1	Community	Frailty; muscle strength (handgrip strength); physical performance (gait speed); physical function (Modified Barthel Index (MBI); quality	Low intrinsic capacity was associated with significant declines in handgrip strength (β=-4.1, 95% CI: -5.67- -2.52), gait speed (β=-0.08, 95% CI: -0.16- -0.007), and physical function (β=-1.2, 95% CI: -2.5- -0.03). Quality of life showed significant decline in low intrinsic capacity (β=-0.053, 95% CI: -0.09- -0.02).

									of life (EuroQoL (EQ)-5D)	
Gonzalez-Bautista, 2020 [18]	France	MAPT	Longitudinal secondary analysis	759	75.2±4.3	483(63.6)	5	Community	Frailty; ADL; IADL	Limited mobility (HR=2.97, 95% CI=1.85–4.76), depressive symptoms (HR=2.07, 95% CI= 1.03–4.19), and visual impairment (HR=1.70, 95% CI: 1.01–2.86) were associated with a higher incidence of frailty. Each additional IC condition demonstrated a positive association with a higher risk of incident frailty, IADL, ADL disability, with risk increased by 47%, 27%, and 23%.
Li, 2021 [25]	China	Not mentioned	Longitudinal study	221	73.7±3.65	156(70.6)	3	Hospital	Disease prognosis	Intrinsic capacity score was independent risk factor for the prognosis of older adults (AUC=0.798, 95% CI: 0.732-0.865).
Liu, 2021 [26]	China	Not mentioned	Longitudinal observational cohort study	212	83.8±4.4	126(59.4)	2	Community	Functional decline (ADL); falls	The AUC of intrinsic capacity for the prediction of functional decline was 0.834 (95% CI: 0.777-0.882); intrinsic capacity showed a slightly predictive value for the falls (AUC=0.834, 95% CI: 0.777-0.881).
Locquet, 2021 [27]	Belgium	SarcoPhAge	prospective cohort study	481	73.4±6.12	289(60.1)	5	Community	Mortality risk	Two satisfactory intrinsic capacity domains appeared to be significantly associated with reduced mortality risk: the satisfactory mobility domain (HR=0.45, 95% CI: 0.26-0.79) and the satisfactory psychological domain (HR=0.56, 95% CI: 1.04-3.09).
Prince,	United	10/66 DRG	Cohort study	17031	74.2(71.3-	10627(62.4)	3 to 5	Community	Incident	One or more declines in

2021 [28]	Kingdom				76.3)				dependence; mortality	intrinsic capacity predicted incident dependence (HR=1.91, 95% CI: 1.69-2.17) and death (HR=1.66, 95% CI: 1.49-1.85).
Sánchez-Sánchez, 2021 [29]	France	INCUR study	Prospective observational study	371	85.91±7.34	263(70.89)	1	Nursing home	Mortality; hospitalization; pneumonia onset; functional status	Intrinsic capacity predicted death (HR=0.33; 95% CI: 0.15-0.73) and functional status (β=0.14; 95% CI: 0.018-0.29). Greater vitality domain of intrinsic capacity was associated with survival (HR=0.84; 95% CI: 0.70-0.99), cognitive domain was associated with decreased odds of hospitalization (HR=0.91; 95% CI: 0.84-0.99) and lower declines in functional status (β=0.04; 95% CI: 0.01-0.07), whereas the locomotion domain was associated with pneumonia incidence (HR=0.84; 95% CI: 0.72-0.98).
Stolz, 2021 [30]	America	Yale Precipitating Events Project Study	Longitudinal cohort study	754	78.4±5.3	506(67.1)	21	Community	Onset of chronic ADL disability; long-term nursing home stay (NHS); mortality	A 1-point lower intrinsic capacity was associated with a 7% increase in the risk of ADL, a 6% increase in the risk of a nursing home stay, and a 5% increase in mortality.
Yu, 2021 [31]	China	MrOS and MsOS (Hong Kong) study	Longitudinal cohort study	3736	72.2(65-98)	1857(49.7)	7	Community	IADL	Intrinsic capacity had a direct effect in predicting incident IADL limitations at the 7-year follow-up (β=-0.21, P<0.001).
Yu, 2021 [32]	China	Mr and MsOS (Hong Kong) study	Prospective cohort study	4000	72.5±5.2	2000(50.0)	2 and 4	Community	Frailty (5-item Cardiovascular Health Study (CHS) frailty phenotype)	Higher scores were associated with a lower risk of incident frailty at both follow-ups (year 2, OR=0.64, 95% CI: 0.59-0.71; year 4, OR=0.64, 95% CI: 0.58-0.71).

Yu, 2021 [33]	China	Not mentioned	Longitudinal study	756	69.3±6.6	518(68.5)	1	Community	Incident disability (ADL and IADL); recurrent falls; hospitalization; emergency department visits; and quality of life (12-item Short Form Health Survey, consisting of physical component summary (PCS) and mental component summary (MCS))	Cognitive decline, limited mobility and depressive symptoms significantly predicted one-year incident IADL disability (ORs=2.74-5.48, 95% CI: 1.51-19.88). Limited mobility, visual impairment and depressive symptoms predicted one-year incident ADL disability (ORs=1.80-3.08, 95% CI: 1.06-5.08). Visual impairment was significantly predictive of one-year incident recurrent falls (OR=2.85, 95% CI: 1.12-7.21). Limited mobility predicted poor PCS (OR=3.03, 95% CI: 1.63-5.66).
Zeng, 2021 [34]	China	Not mentioned	Retrospective observational study	329	≥80 years: 175(53.2)	135(41.0)	1	Hospital	New ADL dependency; new IADL dependency; mortality	Higher intrinsic capacity was associated with decreased risks of 1-year new ADL dependency (OR=0.53, 95% CI: 0.40-0.70) and new IADL dependency (OR=0.76, 95% CI: 0.61-0.95), and 1-year mortality (OR=0.48, 95% CI: 0.31-0.74).
Zhao, 2021 [35]	China	BLSA II	Cohort study	4742	≥65	65-74 years: 2940 (62.0); 75-84 years: 1692 (35.7); ≥85 years: 110 (2.3)	1	Community	Disability (ADL)	A higher impairment in intrinsic capacity domains showed higher odds of incidence ADL disability for impairment in 2 or ≥3 intrinsic capacity domains (OR=2.32 for impairment in ≥3 domains, OR=1.43 for impairment in two domains, AUC =0.685).

Table S4. Measurement tools and methods used for intrinsic capacity

Domain	Measurement methods	Description	References
Locomotion	Chair rise/Chair stand-SPPB	① Repetition of rising from chair measured in seconds (with or without using arm).<br>② Five-times repeated chair stand test (RCST) <12 seconds.<br>③ Perform five chair rises within 14 s.<br>④ Rise from a chair for a total of five times, as quickly as they could, with arms across their chest.	23, 17, 24, 18, 25, 26, 27, 29, 30, 31, 32, 33
	Gait/Walking speed-SPPB	① Time taken to walk 8 feet (2.4m) at usual pace<br>② 3/ 4-metre usual gait speed ≥1.0 m/s<br>③ 5-metre at usual a walking speed of >0.8 m/s.<br>④ Using the best time in seconds to complete a walk along a straight line six meters long in distance.	23, 17, 24, 25, 27, 28, 29, 30, 31, 32, 34
	Standing/ Dynamic balance-SPPB	① Test of standing balance that progressively gets more difficult (side-by-side stand, semi-tandem, full-tandem)<br>② The best time in seconds to complete a narrow walking path (20 cm) over six meters.	23, 17, 25, 27, 29, 30, 31, 32
	the balance subscale of Tinetti Performance-Oriented Mobility Assessment (B-POMA)	B-POMA scores ≤11 indicated poor balance performance	34
	Tinetti Performance-Oriented Mobility Assessment (POMA)	The Tinetti score consists of 13 maneuvers and the score ranges from 0 to 26 (higher is better). The Tinetti test score <24 was considered as an impairment in locomotor capacity.	35
Vitality	Respiratory functioning	① Three readings were taken and the highest technically satisfactory measure of FEV in 1s (FEV1).<br>② The maximum peak expiratory flow value (liter/min) over 3 attempts measured with a Mini-Wright meter.	23, 30
	Handgrip/ Grip/ Muscle strength (kg)	① Three measurements were taken with each hand and the maximum was recorded.<br>② Handgrip strength was measured using a dynamometer.<br>③ Low muscle strength by handgrip strength was defined as a grip strength <28 kg for male participants and <18 kg for female.<br>④ Mean handgrip strength (kg) over 3 readings with a hand-held Chatillon 100 dynamometer.	17, 23, 24, 30, 31, 32, 34
	Body mass index (BMI)	Underweight (<18.5) or obese (≥30) =0; overweight (>25, <30) =0.5 or normal (18.5-25) =1	17
	Abdominal circumference	To nearest 0.1cm.	17
	Mini Nutritional Assessment (MNA)	① A score higher than 17 points (maximum 30 points) indicates no risk of malnutrition.<br>② Malnutrition: weight loss of ≥4.5 kg in the last 3 months.<br>③ MNA score <24 was considered as an impairment in vitality.	17, 24, 27, 28, 35
	Short-Form Mini-Nutritional	① Scores range between 0 and 14, with higher scores indicating better nutritional status.	25, 29, 34

	Assessment (MNA-SF)	② MNA score <11 was considered as an impairment in vitality.	
	Questions about weight loss and appetite loss	① Have you unintentionally lost more than 3 kg over the last 3 months? (weight loss)<br>② Have you experienced loss of appetite? (appetite loss)	18, 26, 33
	Appendicular skeletal muscle mass (ASM)	ASM was measured by dual-engery X-ray absorptiometry (DXA) using Hologic QDR 4500 densitometers.	31, 32
	Biomarkers	Dehydroepiandrosterone (DHEA) and Insulin-like growth factor (IGF-1)	23
Cognition	Time and space orientation plus word recall	-	18
	Recall, verbal and letter tests	① Verbal (semantic) fluency assessed by asking participants to name as many animals as they could think of in 1min.<br>② Delayed verbal memory assessed using lists of nouns presented aurally. Attention assessed using a letter cancellation task.	23
	Sub-parts of Mini-Mental State Examination (MMSE)	Two parts (1) assessment of orientation ability in time (What is the full date today? Where are you now (home, clinic, etc)?) and (2) memory retention capacity (Recall the three words?)	17, 26
	18-item Chinese Mini-Mental Status Examination (CMMSE)	-	24
	Mini-Mental Status Examination (MMSE)	① Cognitive impairment: score Illiteracy ≤ 17 points, primary school ≤ 20 points, junior high school and above ≤ 24 points.<br>② Considered satisfactory when >26 points (out of a maximum of 30 points).	25, 27, 30, 31, 32, 34, 35
	Community Screening Instrument for Dementia (CSI-D)	Scored ≥29.5 were considered to have cognitive capacity, with scores below that threshold identifying “probable dementia”.	28
	Hodkinson’s Abbreviated Mental Test	A 10-item scale spatial and time orientation, memory, and executive function; scores vary from 0 (worse performance) to 10 (best performance).	29
	Short Portable Mental Status Questionnaire (SPMSQ)	The total score ranges from 0 to 10, and cutoff scores for the SPMSQ are 0-2 (intact cognitive function), and 3-10 (cognitive impairment)	33
Sensory	Self-reported vision	①Answering “yes” to any of: “Even if wearing glasses, do you have visual problems to a) distinguish the faces of people in the same room? b) move indoors/outdoors? c) other activities (reading a paper, watching television)?”<br>② Participants were asked to rate their ability to see far, to read and or if they have poor eyesight that interfere with daily activities.<br>③ Participants complained of decreased visual acuity that interfered with normal daily life.	18, 23, 25, 26, 28, 29, 33, 34, 35
	Self-reported hearing	① Answering “sometimes” or “yes” to the question “Do you have difficulty hearing when someone speaks in a whisper?”<br>② Participants complained of hearing loss that interfered with normal daily life.	18, 23, 25, 26, 28, 29, 33, 34, 35
	Self-reported Strawbridge questionnaire	The items for audition and vision were used. Audition is coded from 1 to 12 and vision from 1 to 8, such that the lower the score is, the better the sensory ability.	17
	Jaeger chart (near-vision acuity)	-	30

	Audioscope (hearing impairment)	-	30
	Snellen "Tumbling E" chart	Participants were asked to recognize the direction the "legs" of the E were facing, from the biggest to the smallest line that they could correctly recognize.	31, 32
	Frisby Stereo test (Stereopsis)	Participants were tested at 40 cm away from the plate; if they failed to recognize, they would then view them at 30 cm. This test consists of three transparent plates (1.5, 3, and 6-mm thick) marked with four different pattern squares.	31, 32
Psychological	8-item Center for Epidemiological Studies Depression scale (CES-D-8)	Self-report depression scale, score range: 0-8.	23
	Self-report sleep disturbance	The frequency of delay in falling asleep, inability to stay asleep, waking up tired and disturbed sleep in the previous month	23
	The item "anxiety/depression" of the EuroQol-5D (depression)	3-point Likert scale: (1 = "I am not anxious or depressed", 2 = "I am moderately anxious or depressed", 3 = "I am extremely anxious or depressed")	17
	Two questions of the Center for Epidemiological Studies Depression-CES-D (fatigue)	Self-report on fatigue by two questions: I felt that everything I did was an effort and I could not get going during the past week	17
	10-item Geriatric Depression Scale (GDS-10)	Scores range from 0 to 10, higher values indicating greater levels of depressive symptoms.	29
	15-item Geriatric Depression Scale (GDS-15)	① Answering "yes" to the item 2 of GDS-15 "Have you dropped many of your activities and interests?", or responding "no" to the item 7 of the GDS-15 "Do you feel happy most of the time?"<br>② Over the past two weeks, have you been bothered by: feeling down, depressed or hopeless? little interest or pleasure in doing things?<br>③ The GDS-15 score ≥ 8 indicated a decline in psychosocial functioning.<br>④ The GDS-15 score ≥6 indicated worse psychological performance.<br>⑤ GDS total score ≤4 and if participants did not endorse the modified CES-D items on fatigue.<br>⑥ A score comprised between 12 and 15 indicates severe depression, between 9 and 11 indicates moderate depression, between 5 and 8 indicates mild depression, and between 0 and 4 indicates normal functioning.	18, 24, 25, 26 27, 31, 32, 34, 35
	EURO-D depression scale	12 depression symptoms covered in the EURO-D depression scale.	28
	11-item Center for Epidemiological Studies Depression scale (CES-D-11)	11 items (transformed range = 0-60)	30
	Comprehensive Frailty Assessment Instrument (CFAI)	1 item from CFAI: "Have you felt down in the past week?"	33
	Center for Epidemiologic Studies Depression Scale (CES-D)	1 item from CES-D: "Have you felt that you could not get going?"	33
Continence	Incontinence (urinary, faecal, or both)	Incontinence was established from informant report.	28

- Not described
